# Anti‐Inflammatory Effect of Goat Milk Against Acetic Acid‐Induced Ulcerative Colitis in Rat Model

**DOI:** 10.1002/fsn3.70641

**Published:** 2025-08-08

**Authors:** Shabana Kousar, Maria Arshad, Tahir Zahoor, Muhammad Naeem Faisal, Gholamreza Abdi, Rana Muhammad Aadil

**Affiliations:** ^1^ National Institute of Food Science and Technology University of Agriculture Faisalabad Pakistan; ^2^ Department of Veterinary Science, Institute of Physiology and Pharmacology University of Agriculture Faisalabad Pakistan; ^3^ Department of Biotechnology, Persian Gulf Research Institute Persian Gulf University Bushehr Iran

**Keywords:** antioxidant, colitis, goat milk, intestinal inflammation, oxidative stress, rats

## Abstract

The current study evaluated the protective and antioxidant effects of goat milk, alone and in combination with sulfasalazine (SAZ), against acetic acid (AA)‐induced ulcerative colitis (UC) in Sprague Dawley rats. Thirty rats were divided into five groups with six rats in each group. UC was induced using 3% AA, administered intrarectally (IR) at a dose of 3 mL per rat once daily for the first three consecutive days of the experiment. Goat milk and SAZ treatments were administered orally (p.o.) for 21 days. Group I served as the normal control group and received a basal diet without UC induction. Group II was the positive control group and received 3% AA (3 mL/rat, IR) without any treatment. Group III received AA (IR) and goat milk at a dose of 40 mL/kg body weight (p.o.). Group IV received a combination of goat milk (40 mL/kg body weight, p.o.) and SAZ (250 mg/kg body weight, p.o.) following AA induction. Group V was administered AA (IR) and treated with SAZ alone at a dose of 250 mg/kg body weight (p.o.). Biochemical analysis showed that goat milk, especially in combination with SAZ, significantly reduced malondialdehyde (MDA) levels and elevated antioxidant enzyme levels including superoxide dismutase (SOD), catalase (CAT), and glutathione (GSH) (*p* < 0.05). Hematological parameters such as red blood cell (RBC) counts improved, while elevated white blood cells (WBCs) and lymphocytes were significantly reduced (*p* < 0.05) in the treatment groups. Histopathological examination confirmed attenuated mucosal damage, inflammation, and crypt distortion, with the most pronounced recovery observed in the combination therapy group. The outcome suggested that goat milk, particularly when combined with SAZ, exerts protective effects against AA‐induced colonic inflammation. The anti‐colitis effect may be due to antioxidant and anti‐inflammatory properties of goat milk.

## Graphical abstract

Goat milk was evaluated for its anti‐inflammatory potential. Ulcerative colitis was induced using acetic acid in a rat model. Goat milk significantly reduced colonic inflammation and ulceration. Histological analysis showed marked mucosal healing. Results suggest goat milk as a potential therapeutic in colitis management.

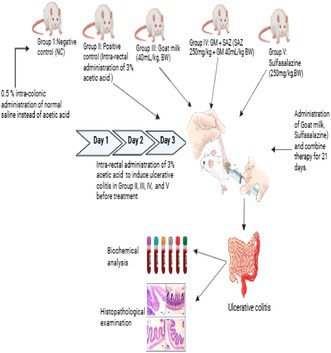

## 1 Introduction

Ulcerative colitis (UC) is a chronic inflammatory condition of the colon, classified under inflammatory bowel disease (IBD) (Azzouz and Sharma 2023; Cellat et al. 2023). Unlike acute colitis, which is often self‐limiting and triggered by infections, chemicals, or radiation, UC is an immune‐mediated condition that requires long‐term medical management (Dulai and Jairath 2018; Li et al. 2023). Although its exact etiology remains unclear, UC is thought to arise from a complex interplay of genetic predisposition, environmental triggers, alterations in gut microbiota, and immune dysregulation (Hassan et al. 2022). The global incidence of UC has been steadily increasing, particularly in industrializing nations, likely due to changing dietary habits and lifestyles (Rai et al. 2022). As a result, UC is now recognized as a significant public health concern due to its rising prevalence, high treatment costs, and impact on patient quality of life (Han et al. 2021).

Current therapeutic strategies for UC include amino‐salicylates such as sulfasalazine (SAZ), corticosteroids, immunosuppressants, and biologics (Guvenc et al. 2019). Although effective in managing inflammation and maintaining remission, these treatments are associated with several adverse effects, including hepatotoxicity, nephrotoxicity, and increased susceptibility to infections when used long‐term (Tunc et al. 2021). Moreover, variable patient responses and the risk of relapse highlight the urgent need for safer, supportive, and more cost‐effective treatment options (Mageed et al. 2024).

Milk is an important and valuable food, which is useful against several diseases (Alorainy et al. 2023; Tariq et al. 2024; Silvi et al. 2024). Among these, goat milk has recently gained attention for its potential therapeutic value in managing inflammatory diseases, including UC (Albarrak and Al‐Sobayil 2024). It is nutritionally rich, providing high‐quality proteins, medium‐chain fatty acids, calcium, and vitamin A nutrients that are essential for intestinal repair and immune modulation (ALKaisy et al. 2023). Compared to cow's milk, goat milk contains lower levels of αs1‐casein and smaller fat globules, which reduce allergenicity and gastrointestinal irritation (dos Santos et al. 2023; Zhao et al. 2023). Goat milk also possesses significant antioxidant and antimicrobial properties because it contains bioactive components such as oligosaccharides, antioxidant peptides, and immunomodulatory compounds, which contribute to its gut‐protective and anti‐inflammatory effects in experimental models (Liu et al. 2021b; Saikia et al. 2022; Qin et al. 2021). Although the general health benefits of goat milk are well recognized, scientific data on its specific effects in UC remain limited (Chen et al. 2020). Given the limitations of existing pharmacological therapies, there is growing interest in evaluating natural and functional foods as complementary or alternative approaches for UC management.

Therefore, the purpose of this study was to investigate the therapeutic potential of goat milk administered alone and in combination with SAZ in an acetic acid (AA)‐induced UC model in rats. The study aimed to assess clinical symptoms, biochemical markers of oxidative stress and inflammation, hematological parameters, and histopathological changes to evaluate the efficacy of goat milk in attenuating colonic inflammation and supporting mucosal healing.

## 2 Materials and Methods

### 2.1 Goat Milk

Fresh goat milk used in this study was sourced from a premium dairy farm located in Faisalabad, Pakistan. Milk was obtained from healthy Beetal goats, a breed known for its high milk yield and nutritional quality. The goats selected for this study had an average body weight of approximately 30 kg and produced around 1.8 L of milk per day.

To ensure milk quality and consistency, the animals were fed a diet formulated in accordance with the National Research Council (NRC) guidelines, targeting a daily milk production of 2 kg with a fat content of approximately 4%. This standardized feeding protocol ensured that the goat milk met specific nutritional and compositional criteria, which are essential for accurately evaluating its potential health benefits (Sikes et al. 2019).

### 2.2 Reagents

All chemicals and reagents used in this study, including AA, glacial metaphosphoric acid, ethylenediaminetetraacetic acid (EDTA), butylated hydroxytoluene (BHT), trichloroacetic acid, tricarboxylic acid, thiobarbituric acid (TBA), 5,5′‐dithiobis‐(2‐nitrobenzoic acid) (DTNB), reduced glutathione (GSH) standard, phosphate buffer, hydrogen peroxide (H_2_O_2_), xanthine oxidase, chloroform, formaldehyde, and normal saline, were obtained from the Food Microbiology and Biotechnology Laboratory at the National Institute of Food Science and Technology, University of Agriculture, Faisalabad, Pakistan. All reagents were originally procured from St. Louis, MO, USA (most commonly referring to Sigma‐Aldrich). The standard drug sulfasalazine (SAZ, 250 mg) used in the treatment protocol was purchased from Mujahid Pharmacy, Jinnah Colony, Faisalabad, Pakistan.

### 2.3 Animal and Experimental Design

Female Sprague Dawley rats, aged 22 weeks and weighing between 180 and 200 g, were procured from the University of Veterinary and Animal Sciences, Lahore, Pakistan. The animals were housed in the animal facility of the Department of Animal Husbandry, University of Agriculture Faisalabad, under standard laboratory conditions. Ethical approval for the study was obtained from the Office of Research, Innovation and Commercialization (ORIC), University of Agriculture Faisalabad, under reference number 1317/ORIC (dated 19 March 2021). All procedures were conducted in accordance with the Pakistan Biosafety Committee (2005), Punjab Biosafety Rules (2014), Animal Health Act (2019), and institutional bioethics protocols.

Before initiating the experiment, the animals were acclimatized for 7 days under controlled environmental conditions, including a 12‐h light/dark cycle, a temperature of 23°C ± 3°C, and relative humidity of 56% ± 2%, with proper ventilation. During the entire study period, rats had free access to a standard pellet diet and fresh water. A total of 30 healthy male Sprague Dawley rats were randomly assigned to five experimental groups, with six rats per group (*n* = 6). UC was induced in all groups except the negative control by intrarectal administration of 3% AA at a volume of 3 mL per rat, once daily for three consecutive days (Days 1–3). AA was delivered using a flexible polyethylene cannula (2‐mm diameter) inserted approximately 8 cm into the rectum, following established protocols (Owusu et al. 2020). After administration, rats were held in a head‐down position for 30 s to ensure retention of the solution.

Group I served as the negative control and received only a basal diet without colitis induction or treatment. Group II was the positive control group, in which UC was induced by AA (3 mL, IR) but no treatment was administered. Group III received goat milk alone at a dose of 40 mL/kg body weight per day, administered orally (p.o.) via gavage. Group IV received a combination therapy consisting of goat milk (40 mL/kg body weight., p.o.) and SAZ at 250 mg/kg body weight (p.o.). Group V received SAZ alone at a dose of 250 mg/kg body weight (p.o.) (Araújo et al. 2017; Rafeeq et al. 2021). All treatments were administered once daily for 21 consecutive days using a flexible oral gavage needle suitable for the animal's body size. Fresh goat milk was collected daily, maintained under sterile conditions, and administered at room temperature. The full experimental plan, including treatment groups and dosing schedules, is summarized in Table 1.

**TABLE 1:** Experimental design and group allocation.

Groups	Treatment description
Group I (negative control)	Received a basal diet only; no ulcerative colitis (UC) induction or treatment was administered.
Group II (positive control)	Received 3% acetic acid (3 mL/rat) intrarectally (IR) once daily for 3 consecutive days to induce UC; no treatment was given.
Group III (goat milk treated)	Induced with UC using 3% acetic acid (3 mL/rat, IR, for 3 days), followed by goat milk (40 mL/kg body weight/day, p.o.) for 21 days.
Group IV (combination therapy)	Induced with UC as above, followed by a combination treatment of goat milk (40 mL/kg body weight/day, p.o.) and sulfasalazine (250 mg/kg body weight/day, p.o.) for 21 days.
Group V (drug control)	Induced with UC as above, followed by sulfasalazine alone (250 mg/kg body weight/day, p.o.) for 21 days.

### 2.4 Ulcer Induction

UC was induced using a well‐established protocol (de Souza Araujo et al. 2016). Three % AA solution (3 mL per rat) was administered intrarectally (IR) once daily on the first three consecutive days of the experiment (Days 1, 2, and 3) to induce colitis (Sanei et al. 2014). AA was delivered using a 2‐mm cannula inserted approximately 8 cm into the rectum. After administration, rats were held in a head‐down position for 30 s to ensure proper retention of the AA within the colon and to minimize immediate leakage. Prior to the first induction, all animals were fasted for 24 h, with continued free access to water.

At the end of the experiment, the rats were anesthetized using chloroform and sacrificed by decapitation (Mageed et al. 2024). The colonic tissues were carefully excised and collected for macroscopic observation, histological examination, and biochemical analysis.

### 2.5 Macroscopic Assessment of the Damaged Colonic Tissues Under Histological Examination

For histopathological evaluation, colon tissues were collected and trimmed into small segments, then fixed in 4% paraformaldehyde for 24 h. The preserved samples were processed through standard paraffin embedding. Tissue sections were sliced, and paraffin was removed by incubating the slides in an oven at 65°C for 2 h. Deparaffinization was performed using xylene, followed by rehydration through a graded ethanol series.

The tissue sections were then stained with hematoxylin and eosin (H&E), dehydrated in ethanol, and cleared again with xylene. Finally, fixation of slides was performed by using resins and cleared with the help of xylene. Histological evaluation was performed under 200× magnification. A quantitative scoring system was used to assess tissue damage based on histological features, including epithelial necrosis, vascular inflammation, leukocyte infiltration, sub‐mucosal edema, and overall tissue architecture disruption (Han et al. 2024). Histological scoring (reference number: 3138) was conducted at the Medical University Faisalabad, Pakistan.

Macroscopic colonic damage was also assessed using a 10‐point scoring scale, as described by Rafeeq et al. (2021). Each colon was longitudinally opened and examined for visible signs of damage, including ulceration, edema, hyperemia, and thickening. The severity of inflammation was scored and is presented in Tables 2 and 3.

**TABLE 2:** Colon damage score by using 10 scale points.

Scores	Description
0	Indicates there is no damage or ulceration
1	Shows there is hyperemia but no ulceration
2	Represents linear ulcer with no inflammation
3	Illustrates linear ulceration but inflammation at one site
4	Describes about ≥ 2 sites where inflammation is present
5	Depicts about ≥ 2 sites where ulceration or inflammation covering portion of more than 1 cm
6–10	The point 6 to 10 express the damage expands more than 2 cm of area of colon

**TABLE 3:** Damage score, weight/length ratio, RBCs, WBCs, platelets, and lymphocytes count across the groups.

Treatment groups	Score	Weight/length ratio	RBC's	WBC's	Platelets	Lymphocytes
NC	0 (0)	85.55 ± 2.60	5.30 ± 0.18^a^	7.81 ± 0.27^c^	2.81 ± 0.10^a^	70.00 ± 2.38^d^
PC	8.5 (6–9)	155 ± 11.50	2.35 ± 0.30	11.87 ± 0.24	0.81 ± 0.13	119.6 ± 5.04
T1	4.5 (3–6)	140.3 ± 5.24	3.90 ± 0.13^c^	8.81 ± 0.30^b^	1.91 ± 0.06^c^	93.00 ± 3.16^b^
T2	4 (2–6)	137.5 ± 4.21	4.50 ± 0.15^b^	8.01 ± 0.27^c^	1.91 ± 0.06^c^	80.00 ± 2.72^c^
SAZ	5.8 (4–5)	147.1 ± 9.50	3.00 ± 0.10^d^	10.51 ± 0.36^a^	1.42 ± 0.05^d^	110.00 ± 3.74ª

*Note:* The score of colonic damage is depicted as in the range of parenthesis with median. The ratio of weight/length showed, all the treatment groups were statistically different from the colitc group (*p* < 0.05). NC, negative control; PC, positive control group; T1, treatment group (goat milk 40 mL/kg of B.W); T2, treatment group (goat milk 40 mL/kg of B.W + SAZ (250 mg/kg of B.W)); SAZ, standard drug‐treated group (SAZ 250 mg/kg of B.W). Values with different letters in the same column (a‐d) are significantly different (*P* < 0.05) from each other.

### 2.6 Biochemical Analysis

Biochemical evaluation of colonic tissue damage involved quantifying oxidative stress and antioxidant markers, including malondialdehyde (MDA), superoxide dismutase (SOD), catalase (CAT), and GSH. The procedures were carried out according to the protocol described by Rafeeq et al. (2021).

Colonic tissues were homogenized in phosphate‐buffered saline using a tissue homogenizer, and the resulting homogenates were centrifuged to obtain clear supernatants for biochemical assays. All analyses were conducted using a UV–visible spectrophotometer under standard laboratory conditions. MDA levels were measured as an indicator of lipid peroxidation and expressed in terms of μmol of hydrogen peroxide degraded per minute per gram of tissue at 25°C. The total GSH content was quantified and reported as nmol/g of wet tissue. SOD and CAT activities were also determined from wet tissue samples and expressed in appropriate enzymatic activity units. These measurements provided insights into the oxidative stress status and antioxidant defense response in the colonic mucosa following colitis induction and treatment.

#### 2.6.1 Measurement of Glutathione (GSH)

GSH, a key intracellular antioxidant, was measured following the method described by Sanei et al. (2014), with slight modifications. The reagents used for sample preparation included a 300 μL precipitating solution composed of 1.67 g glacial metaphosphoric acid, 0.20 g ethylene diamine tetraacetic acid (EDTA), and 30 g sodium chloride (NaCl), dissolved in distilled water.

For the assay, 200 μL of colon tissue homogenate was mixed with 300 μL of distilled water and 300 μL of the precipitating solution in an Eppendorf tube. The mixture was incubated for 5 min at room temperature and then centrifuged at 5000 rpm for 10 min.

To quantify GSH, 200 μL of the resulting supernatant was transferred to a cuvette containing 0.3 M disodium hydrogen phosphate (Na_2_HPO_4_) and 5,5′‐dithiobis‐(2‐nitrobenzoic acid) (DTNB). The absorbance was measured at 412 nm against a reagent blank containing only phosphate buffer (Korkina et al. 2003; Tunc et al. 2021). GSH levels were expressed as nmol per gram of wet tissue.

#### 2.6.2 Measurement of Malondialdehyde (MDA)

The quantity of MDA was measured by thiobarbituric acid reactive species (TBARS) analysis followed by Yagi's method. Isolated colonic tissues were placed in an ice‐cold solution that consisted of 10% TBARS (0.67%) and the solution was heated for 15 min at 90°C. MDA was measured in nmol/mg protein against 532 nm of absorbance.

#### 2.6.3 Super Oxide Dismutase (SOD)

SOD activity was determined according to the method of Fridovich. The assay is based on the enzyme's ability to inhibit the formation of formazan dye, which is generated by the reaction of superoxide radicals with p‐iodonitrotetrazolium violet. Superoxide radicals were produced in the reaction mixture through the enzymatic oxidation of xanthine by xanthine oxidase. The resulting reduction of p‐iodonitrotetrazolium violet formed a colored formazan product, the absorbance of which was measured at 505 nm using a Spectronic UV‐120 spectrophotometer. The reaction mixture consisted of 0.01 M phosphate buffer, 0.05 M substrate solution, and 80 μL of xanthine oxidase. The degree of inhibition of formazan formation was directly proportional to SOD activity, which was expressed as units per milligram of protein (U/mg protein) (Korkina et al. 2003).

#### 2.6.4 Measurement of Catalase (CAT)

CAT activity was measured using the method described by Beutler, as referenced by Korkina et al. (2003). The assay quantifies the enzymatic breakdown of hydrogen peroxide (H_2_O_2_) in the presence of CAT. The reaction mixture consisted of phosphate buffer (pH 7.0), an additional buffer adjusted to pH 8.0, and 10 mM hydrogen peroxide as the substrate. The decrease in absorbance due to the decomposition of hydrogen peroxide was measured spectrophotometrically at 240 nm using a Spectronic UV‐120 spectrophotometer. CAT activity was expressed as units per milligram of protein (U/mg protein), indicating the amount of enzyme required to decompose 1 μmol of H_2_O_2_ per minute under standard conditions.

### 2.7 Hematological Analysis of Blood

Hematological parameters, including red blood cells (RBCs), white blood cells (WBCs), platelets, and lymphocytes, were evaluated according to the protocol described by Abd Majid et al. (2020). Whole blood samples were collected from each rat into EDTA‐coated tubes to prevent clotting. Complete blood counts (CBC) were performed in the Molecular Laboratory, University of Agriculture Faisalabad, using an automated hematology analyzer. Blood samples were processed immediately after collection to ensure accuracy.

### 2.8 Statistical Analysis

All results were expressed as mean ± standard error of the mean (SEM). Statistical significance between group means was evaluated using one‐way analysis of variance (ANOVA), followed by Tukey's post hoc test for multiple comparisons. Data analysis was performed using GraphPad Prism version 10, and differences were considered statistically significant at *p* < 0.05 (de Souza Araujo et al. 2016).

## 3 Results

### 3.1 Monitoring of Clinical Symptoms and Disease Severity

Body weight loss and the presence of blood in the stool were monitored throughout the experimental period as indicators of disease severity. Rats were weighed on Days 1, 8, 15, and 20 to assess the effects of UC induction and subsequent treatments on body weight changes.

As shown in Table 3, significant differences (*p* < 0.05) in body weight were observed across alternate days, particularly on Days 15 and 21, indicating that the treatments had a notable impact on weight variation.

Rats in the goat milk‐treated group exhibited initial weight loss during the first week, followed by gradual weight gain in the second week, in contrast to the negative control group. This pattern suggests that the early weight loss was due to the acute phase of UC, while the subsequent weight gain reflected partial disease remission by the end of the study period.

Furthermore, treatment groups experienced less weight loss compared to the standard drug group. This effect may be attributed to the oligosaccharides present in goat milk, which likely enhanced feed intake by mitigating the appetite‐suppressing effects of AA (Lautenschlager et al. 2023).

### 3.2 Effect of Goat Milk on Macroscopic Assessment of Colonic Damage

Colonic injury was induced using 3 mL of 3% AA administered IR, a well‐established method for inducing experimental colitis (Rafeeq et al. 2021). The severity of colonic damage was evaluated based on key macroscopic indicators, including leukocyte infiltration, hemorrhage, deep tissue inflammation, ulceration (red or black spots), and mucosal discoloration.

The noncolitic control group, which received saline instead of AA, exhibited no signs of inflammation or tissue damage. In contrast, the colitis‐induced group displayed extensive mucosal injury, an increased weight/length ratio of the colon, and severe inflammation.

Goat milk treatment significantly reduced the severity of colonic damage compared to the colitis‐induced group (*p* < 0.05). Notable improvements included lower weight/length ratios and reduced bowel wall thickening (*p* < 0.05).

Interestingly, the group receiving a combination of goat milk and SAZ showed even greater improvements than the group treated with SAZ alone (*p* < 0.01), suggesting a possible synergistic effect. In this combination group, tissue edema and inflammatory cell infiltration were markedly reduced compared to the SAZ‐only group.

**FIGURE 1:**
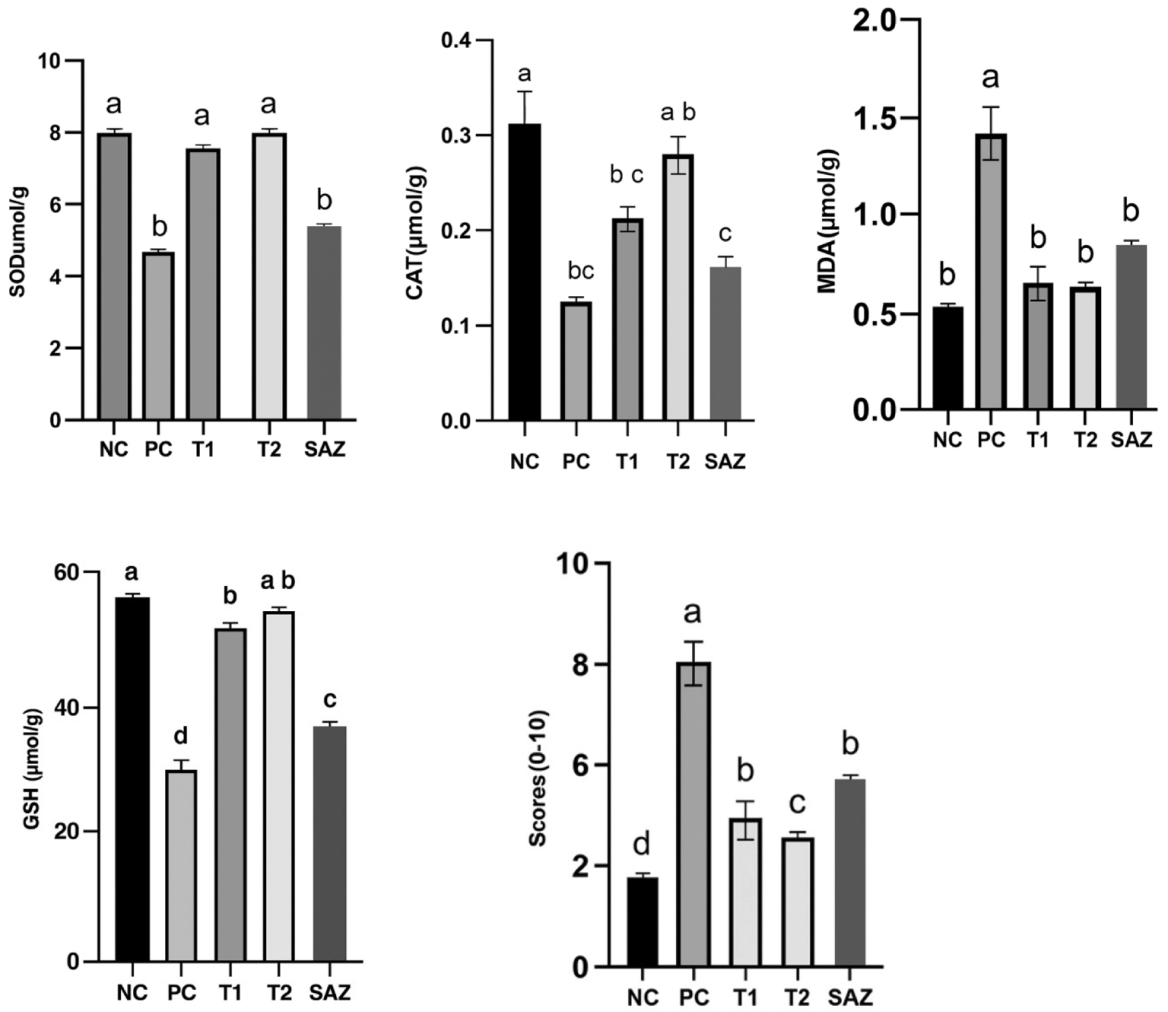
Effect of goat milk (GM) on superoxide dismutase (SOD), catalase (CAT), glutathione (GSH), malondialdehyde (MDA), and colonic damage scores in acetic acid‐induced ulcerative colitis rats. The experimental groups included: negative control (NC), positive control (PC), T1 group (treated with goat milk, 40 mL/kg body weight), T2 group (treated with a combination of goat milk and SAZ, 40 mL/kg and 250 mg/kg body weight, respectively), and drug‐treated group (SAZ, 250 mg/kg body weight). Different lowercase letters (a, b, c, etc.) above the bars indicate statistically significant differences among the groups (*p* < 0.05).

Table 3 and Figure 1 present detailed comparisons of colonic damage scores and weight/length ratios across all experimental groups.

### 3.3 Evaluation of Goat Milk's Effect on MDA, CAT, GSH, and SOD

To assess oxidative stress and the antioxidant defense system in colitis‐induced rats, levels of MDA, GSH, CAT, and SOD were measured. MDA levels were significantly elevated in the colitis‐induced group compared to the negative control (*p* < 0.01), indicating enhanced lipid peroxidation and oxidative stress. Treatment with goat milk or SAZ individually led to a significant reduction in MDA levels (*p* < 0.05), suggesting their capacity to counter oxidative damage.

Notably, the group receiving the combination of goat milk and SAZ exhibited the most pronounced reduction in MDA levels (*p* < 0.01) compared to the SAZ‐only group. This decline in MDA was accompanied by a significant increase in antioxidant enzyme activities, including GSH, CAT, and SOD (*p* < 0.01), indicating a strong antioxidant response.

The combination therapy group (goat milk + SAZ) demonstrated significantly higher antioxidant enzyme levels than the SAZ‐only group (*p* < 0.01), suggesting a synergistic or additive effect in mitigating oxidative stress. The enhanced antioxidant activity observed in this group underscores the potential of goat milk, particularly its bioactive oligosaccharides, in promoting colonic healing and supporting disease remission.

Overall, the treatment groups exhibited varying degrees of antioxidant response, with the combination therapy showing the most significant effect. Statistical comparisons confirmed a significant difference between goat milk alone and the combination treatment group (*p* < 0.05), reinforcing the role of goat milk in alleviating oxidative stress.

Table 4 and Figure 1 present the levels of oxidative stress markers (MDA) and antioxidant enzymes (GSH, CAT, and SOD) across the experimental groups.

**TABLE 4:** Effect of goat milk on GSH, CAT, SOD, and MDA across the different treatment groups.

Treatment groups	GSH(nmol/g)	CAT(μmol/g)	SOD(μmol/g)	MDA(μmol/g)
NC	55.64 ± 1.89^a^	0.24 ± 0.01ᵃ	6.61 **±** 0.22ᵃ	0.55 ± 0.03ᵇ
PC	29.14 ± 3.02	0.11 ± 0.00	3.86 ± 0.5	1.41 ± 0.25
T1	50.06 ± 1.70ᵇ	0.19 ± 0.01ᵇ	5.54 ± 0.19ᵇ	0.70 ± 0.02^c^
T2	55.05 ± 1.87ᵃ	0.23 ± 0.01ᵃ	6.94 ± 0.24ᵃ	0.62 ± 0.02^d^
SAZ	33.64 ± 1.14^c^	0.14 ± 0.02^c^	4.35 ± 0.11^c^	0.82 ± 0.03ᵃ

*Note:* Mean values with the same alphabet differ nonsignificantly with others (*p* < 0.05). NC, negative control; PC, positive control group; T1, treatment group (goat milk 40 mL/kg of B.W); T2, treatment group (goat milk 40 mL/kg of B.W + SAZ (250 mg/kg of B.W)); SAZ, standard drug‐treated group (SAZ 250 mg/kg of B.W). Values with different letters in the same column (a‐d) are significantly different (*P* < 0.05) from each other.

### 3.4 Hematological Analysis

In the colitis‐induced group, red blood cell (RBC) counts were significantly reduced (*p* < 0.01), likely due to colonic bleeding and inflammation‐associated anemia. Conversely, white blood cell (WBC) and lymphocyte counts were markedly elevated (*p* < 0.01) compared to the control group, reflecting the inflammatory response characteristic of UC. Colitis is also known to increase platelet activation and aggregation, which may contribute to thrombocytopenia. In our study, platelet counts were significantly lower in colitis‐induced rats compared to controls (*p* < 0.05), indicating hematological disturbances related to disease progression.

**FIGURE 2:**
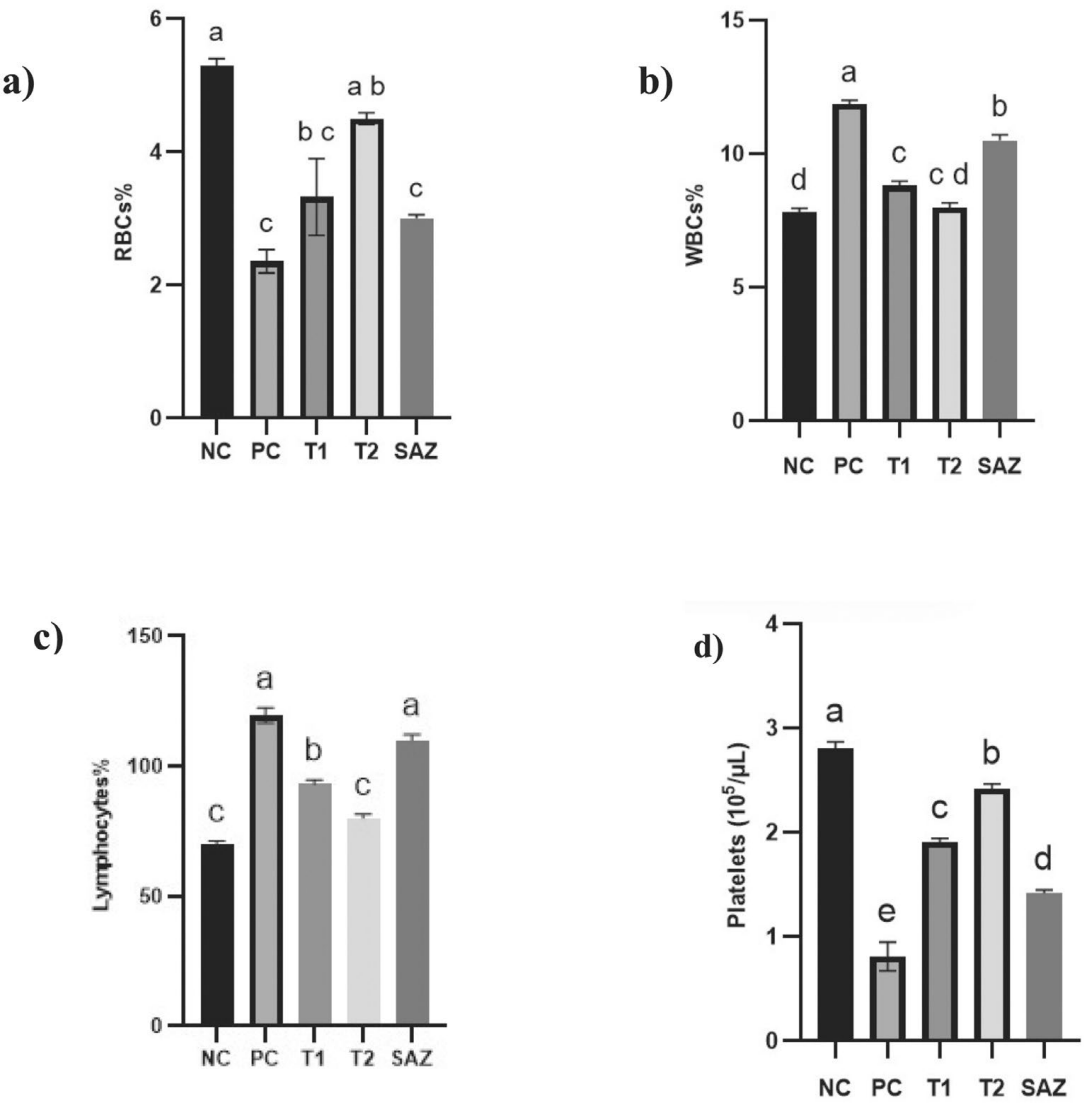
Effect of goat milk (GM) and sulfasalazine (SAZ) on red blood cells (RBCs), white blood cells (WBCs), lymphocytes, and platelet levels in an acetic acid‐induced ulcerative colitis rat model. The experimental groups included: negative control (NC), positive control (PC), T1 group (treated with goat milk, 40 mL/kg body weight), T2 group (treated with a combination of goat milk and sulfasalazine, 40 mL/kg and 250 mg/kg body weight, respectively), and the standard drug‐treated group (SAZ, 250 mg/kg body weight). Groups labeled with different lowercase alphabetical letters (a, b, c, etc.) indicate statistically significant differences (*p* < 0.05). GM, goat milk; RBCs, red blood cells; SAZ, sulfasalazine; WBCs, white blood cells.

After 21 days of treatment, all treated groups showed significant improvement in hematological parameters. Both the goat milk‐treated group and the combination therapy group (goat milk + SAZ) exhibited a notable increase in RBC counts (*p* < 0.05) compared to the SAZ‐only group. The combination therapy group demonstrated the greatest recovery in RBC levels, suggesting a potential synergistic effect of goat milk with SAZ. Additionally, both WBC and lymphocyte counts were significantly reduced in the goat milk and combination therapy groups (*p* < 0.01), indicating a marked attenuation of colitis‐induced inflammation. The combination group showed the most substantial decrease in WBC levels, supporting the enhanced anti‐inflammatory effect of goat milk when used alongside SAZ. Platelet counts also improved significantly in both treatment groups (*p* < 0.05), with the combination therapy group exhibiting the highest restoration of platelet levels. These findings suggest that goat milk supplementation aids in reducing systemic inflammation and restoring hematological balance, potentially mitigating the risk of thrombocytopenia and disease progression. Table 3 and Figure 2 illustrate the comparative effects of the different treatment regimens on hematological parameters.

### 3.5 Histopathological Examination of the Colonic Tissue

Histopathological evaluation of colonic tissue revealed marked differences among the experimental groups. In the colitis‐induced (UC) group, severe epithelial disruption, crypt loss, extensive leukocyte infiltration, hemorrhage, vasodilation, and submucosal edema were observed, confirming the severity of AA‐induced colonic damage. In contrast, the goat milk‐treated group exhibited mild inflammatory cell infiltration, preservation of epithelial architecture, reduced hemorrhage, and less vasodilation, indicating a protective effect of goat milk against colitis‐induced tissue injury.

The combination therapy group (goat milk + SAZ) demonstrated moderate leukocyte infiltration, vasodilation, crypt congestion, and submucosal blood stasis. Despite the presence of some inflammation, the overall colonic architecture was better preserved than in the drug‐treated group, suggesting a synergistic effect between goat milk and SAZ in reducing tissue damage. The SAZ‐only group showed relatively intact colonic morphology with discrete polymorphonuclear cell infiltration in the submucosal layer and lamina propria. However, the crypt structure remained slightly compromised, indicating only partial recovery compared to the combination group.

As expected, the negative control group maintained normal colonic histology, with intact epithelium, well‐organized crypts, and no signs of inflammation or hemorrhage. Additionally, platelet analysis revealed a significant increase in the combination therapy group, which exhibited the highest mean platelet count compared to the SAZ‐only group (*p* < 0.05). This supports the idea that the combined treatment not only reduces inflammation but also helps restore hematological balance. Overall, the combination therapy group demonstrated improved histological preservation and mucosal healing compared to all other groups, further highlighting the therapeutic potential of goat milk alone or in conjunction with SAZ for managing colitis.

**FIGURE 3:**
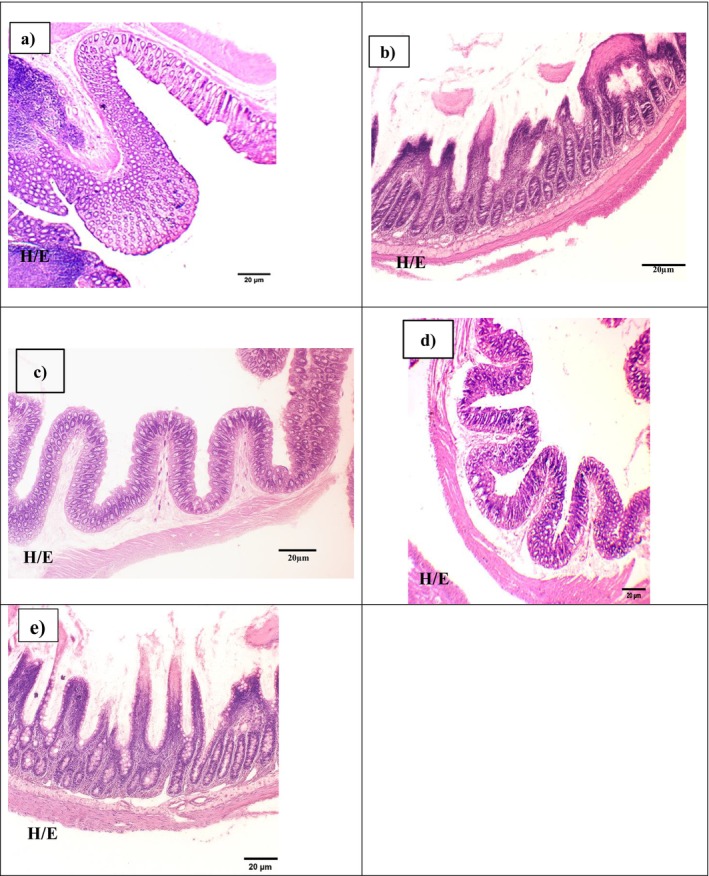
Histopathological examination of colon tissue (H&E staining, 10× magnification): Representative photomicrographs of rat colon sections from the following experimental groups: (a) NC, negative control group; (b) PC, positive control group (acetic acid‐induced colitis); (c) T1, treated with goat milk (40 mL/kg body weight); (d) T2, Co‐treated with goat milk (40 mL/kg body weight) and sulfasalazine (SAZ, 250 mg/kg body weight); and (e) SAZ, standard drug group treated with sulfasalazine alone (250 mg/kg body weight). Tissues were stained with hematoxylin and eosin (H&E) to evaluate mucosal structure, goblet cell preservation, and inflammatory infiltration. Colon tissues were stained with H&E, hematoxylin and eosin for microscopic evaluation. Different lowercase letters indicate statistically significant differences between groups (*p* < 0.05).

Figure 3 illustrates representative histological findings across all experimental groups.

## 4 Discussion

The gut microbiota plays a vital role in the pathophysiology of colitis, a condition characterized by inflammation of the colon (Sanei et al. 2014). The inflammatory process in colitis is closely linked to increased oxidative stress and the excessive production of reactive oxygen species (ROS), as observed in experimental models (Mecocci et al. 2022; Rehman et al. 2022). Conventional treatments, such as antibiotics, often disrupt microbiota composition, leading to potential side effects. In contrast, goat milk has emerged as a promising alternative for modulating gut microbiota and improving colonic health due to its anti‐inflammatory and antioxidant properties. These properties contribute to reducing ROS levels, inducible nitric oxide synthase (iNOS) and pro‐inflammatory mediators while enhancing antioxidant enzyme activity. This dual action of modulating gut microbiota and reducing oxidative stress could be key to its therapeutic potential in colitis management (De Ciucis et al. 2023).

Our study demonstrated that goat milk administration significantly reduced macroscopic colonic damage and inflammation, as observed through histological examination. These findings align with previous research that linked dietary intervention with modulation of gut microbiota and inflammation (De Ciucis et al. 2023). However, unlike previous studies emphasizing general gut health, our findings specifically show that goat milk inhibited abnormal cell infiltration in the colonic mucosa, as indicated by reduced MDA levels (El‐Desoky Mohamady et al. 2022). As MDA acts as a chemotactic mediator promoting leukocyte infiltration, these findings further support the role of oxidative stress in the pathogenesis of UC (Silva et al. 2023). While previous studies reported the antioxidant potential of goat milk, our findings strengthen this evidence by showing its direct effect on oxidative stress and inflammation, as reflected by reduced levels of MDA and TNF‐α (Ansari et al. 2021). These findings offer new insight into the mechanism of goat milk and support its role in alleviating colonic inflammation.

GSH plays a vital role in protecting against oxidative stress and serves as a key indicator of the antioxidant defense system's integrity (Han et al. 2021). Our findings showed a significant decrease in GSH levels in AA‐induced colitis rats, which were restored to near‐normal after goat milk administration. This supports earlier research indicating that GSH contributes to mucosal healing (Freire et al. 2017).

Previous studies have identified goat milk as a rich source of antioxidants, particularly cysteine, which is essential for GSH synthesis (Chen et al. 2020). Our findings advance this understanding by showing that goat milk supplementation not only restored GSH levels but also enhanced GSH peroxidase activity, strengthening the body's defense against oxidative stress. These results provide additional mechanistic support for its therapeutic potential in colitis management (Lautenschlager et al. 2023).

Beyond its antioxidant properties, goat milk has been reported to influence gut microbiota composition. Although our study did not directly assess microbial populations, the observed reduction in colonic inflammation following goat milk supplementation may be partially explained by the prebiotic effects of goat milk oligosaccharides (gMOS), as supported by earlier research (Silva et al. 2023). gMOS have been shown to modulate the immune system, inhibit pathogenic bacteria, and enhance gut barrier function. Additionally, they are known to increase populations of beneficial microbes, such as *Bifidobacteria*, and promote the production of short‐chain fatty acids (SCFAs), including acetate, propionate, and butyrate, which contribute to intestinal health and reduced inflammation (van Leeuwen et al. 2020). While we did not evaluate gMOS content or SCFA levels in this study, our findings are in line with the known physiological effects of these bioactive milk components.

These metabolites, produced through fiber fermentation by gut microbiota, support gut barrier function, regulate glucose metabolism, and modulate inflammation. This study found that goat milk supplementation significantly increased SCFA's levels (Han et al. 2021). Moreover, we confirmed that SCFA's absorption in the colon enhanced the immune response and decreased inflammation, aligning with prior studies emphasizing the role of SCFAs in reducing the risk of UC (Zhu et al. 2019; Liu et al. 2021b). However, unlike general dietary interventions that increase SCFA levels, our study specifically highlighted goat milk's ability to maintain SCFA balance while improving gut microbiota composition, providing further insights into its unique gut‐protective properties.

Interestingly, our findings revealed that goat milk was as effective as SAZ in reducing colonic inflammation and oxidative stress (Anwer et al. 2020). However, unlike SAZ, which is known to negatively impact gut microbiota diversity, goat milk not only reduced inflammation but also preserved gut microbiota homeostasis. This distinction is particularly important, as long‐term antibiotic use can lead to gut dysbiosis, whereas goat milk offers both therapeutic and microbiome‐supporting benefits (Agustin et al. 2024; Arief et al. 2024). Although the findings are promising, they suggest that goat milk may offer supportive benefits alongside SAZ in long‐term colitis management. However, as this is a preclinical animal study, its potential as an alternative requires further validation in human trials (Zhang et al. 2024). Additionally, in line with previous reports, our study showed that goat milk administration significantly improved systemic health markers, including RBC, WBC, lymphocytes, and platelet count in colitis‐induced rats, supporting its overall physiological benefits (Liu et al. 2021a).

Despite these promising findings, our study has certain limitations. First, while we observed significant improvements in gut microbiota composition, our analysis was limited, and a more comprehensive evaluation using next‐generation sequencing would provide deeper insights into microbial shifts. Secondly, as this study was conducted using a UC animal model, future research should explore the long‐term therapeutic potential of goat milk and evaluate whether its protective effects are sustained over time. The results confirmed goat milk's effectiveness in the animal model; clinical trials are required to explore its effectiveness and safety aspects in the human population.

## 5 Conclusion

Goat milk has emerged as a potential therapeutic option for UC, as it contains vital bioactive components including peptides, high‐quality proteins, unsaturated fats, conjugated linoleic acids, and oligosaccharides. These compounds are believed to contribute to its antioxidant and anti‐inflammatory properties, helping protect the intestine by reducing inflammation and enhancing antioxidant levels. Although these findings support the potential role of goat milk in UC management, the precise mechanisms underlying its anti‐inflammatory effects remain unclear. Therefore, further advanced research is needed to fully elucidate and validate the therapeutic potential of goat milk in UC and to clarify the mechanisms responsible for its protective effects.

## Author Contributions

**Shabana Kousar:** conceptualization (equal), data curation (equal), methodology (equal), writing – original draft (equal), writing – review and editing (equal). **Maria Arshad:** investigation (equal), writing – review and editing (equal). **Tahir Zahoor:** resources (equal), supervision (equal), validation (equal), writing – review and editing (equal). **Muhammad Naeem Faisal:** resources (equal), writing – review and editing (equal). **Gholamreza Abdi:** conceptualization (equal) writing – review and editing (equal). **Rana Muhammad Aadil:** resources (equal), supervision (equal), writing – review and editing (equal).

## Ethics Statement

The animal‐based study was approved by the Pakistan Biosafety Committee 2005, Punjab Biosafety Rules 2014, Animal Health Act 2019, and Bioethics Protocol (D#1317/ORIC dated 19‐03‐2021) UAF, Pakistan.

## Conflicts of Interest

The authors declare no conflicts of interest.

## Data Availability

All the derived data supporting the findings of this study are used in this manuscript.
